# Factors associated with breastfeeding cessation in nursing mothers in a peer support programme in Eastern Lancashire

**DOI:** 10.1186/1471-2431-10-3

**Published:** 2010-01-27

**Authors:** Gabriel Agboado, Elaine Michel, Elaine Jackson, Arpana Verma

**Affiliations:** 1 NHS Blackpool, Blackpool Stadium, Seasiders Way, Blackpool, FY1 6JX, UK; 2 NHS Tameside and Glossop, New Century House, Windmill Lane, Denton, M34 2GP, UK; 3 Little Angels, 13-15 Railway Road, Darwen, BB3 2RG, UK; 4 Manchester Urban Collaboration on Health, Manchester Academic Health Sciences Centre, Stopford Building, University of Manchester, Manchester, M13 9PT, UK

## Abstract

**Background:**

The UK has one of the lowest breastfeeding rates worldwide and in recent years the Government has made breastfeeding promotion one of its priorities. The UNICEF UK Baby Friendly Initiative is likely to increase breastfeeding initiation but not duration. Other strategies which involve provision of support for breastfeeding mothers in the early weeks after birth are therefore required to encourage UK mothers to breastfeed for the recommended duration. This paper examines the effects of maternal socio-demographic factors, maternal obstetric factors, and in-hospital infant feeding practices on breastfeeding cessation in a peer support setting.

**Methods:**

Data on mothers from Blackburn with Darwen (BwD) and Hyndburn in Eastern Lancashire who gave birth at the Royal Blackburn Hospital and initiated breastfeeding while in hospital were linked to the Index of Multiple Deprivation (IMD). The data were analysed to describe infant feeding methods up to 6 months and the association between breastfeeding cessation, and maternal factors and in-hospital infant feeding practices.

**Results:**

The mean breastfeeding duration was 21.6 weeks (95% CI 20.86 to 22.37 weeks) and the median duration was 27 weeks (95% CI 25.6 to 28.30 weeks). White mothers were 69% more likely to stop breastfeeding compared with non-White mothers (HR: 0.59; 95% CI, 0.52 to 0.67 [White mothers were the reference group]). Breastfeeding cessation was also independently associated with parity and infant feeding practices in hospital. There were no significant associations between breastfeeding cessation and marital status, mode of delivery, timing of breastfeeding initiation and socio-economic deprivation.

**Conclusion:**

In this study ethnicity, parity and in-hospital infant feeding practices remained independent predictors of breastfeeding cessation in this peer support setting. However other recognised predictors such as marital status, mode of delivery, timing of breastfeeding initiation and socio-economic deprivation were not found to be associated with breastfeeding cessation.

## Background

The UK has one of the lowest breastfeeding rates worldwide [1] and ranked the second lowest among 32 countries in the WHO European Region with a breastfeeding rate at 6 months of 21% in 2000 [2]. Only a modest increase was observed by the 2005 Infant Feeding Survey which recorded a breastfeeding rate at 6 months of 25% [3]. Conversely, some other developed European countries, such as the Scandinavia and Germany have high breastfeeding initiation and continuation rates [4].

In recent years the UK Government has made breastfeeding promotion one of its priorities [5-7] and has amended the breastfeeding and weaning recommendations to recommend that infants should be exclusively breastfed for the first 6 months of life [8] following the revision of the WHO guidelines [9,10]. Most recently the government proposed the Single Equality Bill under which women would get the legal right to breastfeed in public with the aim of boosting breastfeeding prevalence [11]. Some have observed that the UNICEF UK Baby Friendly Initiative is likely to increase breastfeeding initiation but not duration and suggested that other strategies which involve the provision of support for breastfeeding mothers in the early weeks after birth are required to encourage UK mothers to breastfeed for the recommended duration [12].

In 2004 Little Angels, a social enterprise based in Darwen (in Eastern Lancashire), started a collaborative project based on the WHO Ten Steps for Successful Breastfeeding [13] with the Royal Blackburn Hospital, a Baby Friendly Hospital in Lancashire, to support nursing mothers to breastfeed longer. From 2004 to 2006 they helped increase the breastfeeding rates at 6-8 weeks and 7-9 months from 20% to 56% and 4% to 13% respectively [14]. The initiative received the "Award for practices to promote the involvement of women in providing local maternity services" by the All Party Parliamentary Group (APPG) on Maternity [15]. In the Department of Health's 2007 review of progress against the National Service Framework for Children, Young People and Maternity Services, Little Angels were cited as a case study of good practice [16].

The effectiveness of peer support programmes in improving breastfeeding outcomes has been established [4,17-19]. However breastfeeding duration depends on a number of determinants some of which may be modifiable. Knowledge about the type and importance of these determinants in peer support programmes is essential for building effective breastfeeding promotion programmes [20]. Maternal socio-demographic and obstetric factors such as ethnicity [21-23], parity [24-28], marital status [24-28], mode of delivery [24,25] and socio-economic status [3,4,24,29], and infant feeding practices [13,29-33] are important predictors of breastfeeding cessation. The aim of this study is to identify maternal socio-demographic and obstetric factors, and in-hospital infant feeding practices that are associated with breastfeeding cessation among the mothers supported by Little Angels.

## Methods

### The data

Little Angels run a peer support programme which relies on trained peer support workers. They provide 24-hour telephone support to the nursing mothers in breastfeeding difficulties in addition to regular home visits. Information on feeding methods is collected at 6 weeks, 17 weeks, 6 months, and 9 months. Date of stopping breastfeeding and reasons for stopping are some of the additional information collected.

This study used data from mothers on Little Angels' database and covered all mothers resident in Blackburn with Darwen (BwD) and Hyndburn in East Lancashire who gave birth at the Royal Blackburn Hospital and initiated breastfeeding while in hospital. The database was anonymised and held 2,801 records at the time of the study. Records of mothers with babies aged at least 6 months on 30^th ^May 2007 and with valid postcodes were extracted for analyses. In all 2,107 records were identified meeting these criteria. The main bulk of the data retrieved were for mothers who gave birth between January 2005 and December 2006. Thirteen records were for mothers who gave birth in 2004. Ethical approval was sought from the Cumbria and Lancashire Committee but it was considered not required for this study.

The socio-economic status of the mothers were obtained by linking the data to the 2004 Index of Multiple Deprivation (IMD) [34]. The resultant dataset was analysed to describe infant feeding patterns and the association between breastfeeding cessation, and maternal socio-demographic factors, maternal obstetric factors, and in-hospital infant feeding practices (see table 1 for the list of the factors).

**Table 1:** Predictor variables considered for the study

Maternal socio-demographic and obstetric factors	Borough of residence
	Ethnicity
	Marital status
	Parity
	Mode of delivery
	Socio-economic deprivation as measured by the Index of Multiple Deprivation scores.
**Infant feeding practices in hospital**	The timing of breastfeeding initiation after delivery Infant formula-feeding status in hospital Teat-giving status in hospital

The durations of breastfeeding, in weeks, were calculated from babies' dates of birth and dates of breastfeeding cessation stated by the mothers. Where the dates of breastfeeding cessation were not known the feeding methods at 6 weeks, 17 weeks and 6 months were used to estimate the durations. 1,129 (53.6%) of the records did not have dates of breastfeeding cessation of which 856 (75.8%) had entries indicating that the mothers were breastfeeding, at least, up to 6 months.

### Outcomes

This study tested the hypothesis that breastfeeding cessation among the mothers was associated with maternal factors and in-hospital infant feeding practices. The main outcome measures were the mean and median breastfeeding durations in weeks, because the breastfeeding duration data was negatively skewed, and hazard ratios (HRs) of stopping breastfeeding associated with the variables of interest.

The World Health Organization (WHO) has defined three types of breastfeeding namely, exclusive breastfeeding, breastfeeding, and predominant breastfeeding [35]. In this study we used exclusive breastfeeding and breastfeeding as defined by the WHO.

### Statistical analyses

The significance level for all the analyses was set at 5%. Chi-square and Fisher's exact tests were used to test statistical associations for bivariate analyses. Survival analyses techniques were used to estimate the duration of breastfeeding and identify breastfeeding cessation predictors because they provide a good understanding of breastfeeding behaviour over time [27]. Kaplan-Meier (KM) survival analyses were carried out to describe breastfeeding duration by the variables of interest. To quantify the association between these variables and breastfeeding cessation Cox proportional hazards model was used because it is a more powerful statistical technique than single-point prevalence estimates [27]. To identify variables independently associated with breastfeeding cessation Cox multivariate regression analyses were carried out with all the variables included in the model iteratively using the model analyses based on the likelihood ratio test [36]. Variables which did not significantly contribute to the fitness of the model were dropped until the best model was obtained containing ethnicity, parity, formula-feeding and teats-giving status in hospital as predictor variables. In all 2,074 out of 2,107 records were included in the final model. The results from the final multivariate model were reported as the adjusted HRs. StatsDirect (Version 2.6.3) was used for the analyses.

## Results

### Maternal socio-demographic and obstetric characteristics and in-hospital infant feeding practices

Table 2 shows the distribution of maternal socio-demographic and obstetric characteristics. 1,606 (76.2%) of the mothers were from BwD while 501 (23.8%) were from Hyndburn. Whites were the predominant ethnicity recorded overall (48.0%). They constituted 76.0% among Hyndburn mothers while among BwD mothers they formed 39.1% (p < 0.0001). More Hyndburn mothers were from areas with IMD scores in the 1^st ^and 2^nd ^quartile (35.9% and 31.1% respectively) than those from BwD (22.5% and 22.4% respectively; p < 0.0001). 96.7% of the mothers had partners, 42.7% were primiparae and 73.3% had vaginal deliveries; there were no statistically significant inter-borough differences in the distribution of these factors.

**Table 2:** Distribution of maternal socio-demographic and obstetric characteristics.

Variable	Total No (%)	BwD No (%)	Hyndburn No (%)	P value
**Borough of residence**				
Both	2,107 (100%)	1,606 (76.2%)	501 (23.8%)	-
BwD	1,606	1,606	-	-
Hyndburn	501	-	501	-
**Ethnicity**				
White	1,003 (48.0%)	622 (39.1%)	381 (76.0%)	< 0.0001
Non-white	1,088 (52.0%)	968 (60.9%)	120 (24.0%)	< 0.0001
▪ Indian	496 (23.7%)	489 (30.8%)	7 (1.4%)	< 0.0001
▪ Pakistani	503 (24.1%)	403 (25.3%)	100 (20.0%)	0.0139
▪ All Blacks	25 (1.2%)	24 (1.5%)	1 (0.2%)	0.0097
▪ Others^Ψ^	64 (3.1%)	52 (3.3%)	12 (2.4%)	0.3043
Total valid data	2,091 (100.0%)	1,590 (100.0%)	501 (100.0%)	-
Missing data	16	16	0	0.0094
**Marital status**				
Mothers with partners	2,030 (96.7%)	1,551 (97.0%)	479 (95.8%)	0.1557
Single mothers	69 (3.3%)	48 (3.0%)	21 (4.2%)	0.1557
Total valid data	2,099 (100.0%)	1,599 (100.0%)	500 (100.0%)	-
Missing data	8	7	1	0.4498
**IMD quartile**				
1st quartile	542 (25.7%)	362 (22.5%)	180 (35.9%)	< 0.0001
2nd quartile	516 (24.5%)	360 (22.4%)	156 (31.1%)	< 0.0001
3rd quartile	526 (25.0%)	477 (29.7%)	49 (9.8%)	< 0.0001
4th quartile	523 (24.8%)	407 (25.3%)	116 (23.2%)	0.3221
Total valid data	2,107 (100.0%)	1,606 (100.0%)	501(100.0%)	-
Missing data	0	0	0	-
**Parity**				
Primips	897 (42.7%)	675 (42.1%)	222 (44.3%)	0.1952
Multips	1,206 (57.3%)	927 (57.9%)	279 (55.7%)	0.1952
▪ 2^nd ^births	153 (7.3%)	109 (6.8%)	44 (8.8%)	0.1358
▪ 3^rd ^births	521 (24.8%)	392 (24.5%)	129 (25.7%)	0.5580
▪ 4^th ^births	296 (14.1%)	242 (15.1%)	54 (10.8%)	0.0152^◆^
▪ 5^th ^births	155 (7.4%)	125 (7.8%)	30 (6.0%)	0.1759
▪ 6^th ^- 9^th ^births	82 (3.9%)	60 (3.7%)	22 (4.4%)	0.5129
Total valid data	2,103 (100.0%)	1,602 (100.0%)	501 (100.0%)	-
Missing data	4	4	0	0.3112
**Mode of delivery**				
Vaginal	1,537 (73.3%)	1,183 (74.1%)	354 (70.8%)	0.1333
Operative delivery	560 (26.7%)	414 (25.1%)	146 (29.2%)	0.1484
▪ Caesarean	371 (17.7%)	285 (17.8%)	86 (17.2%)	0.7377
▪ Instrumental	189 (9.0%)	129 (8.1%)	60 (12.0%)	0.0074
Total valid data	2,097 (100.0%)	1,597 (100.0%)	500 (100.0%)	-
Missing data	10	9	1	0.2989

^Ψ^Chinese, Bangladeshi, other ethnic groups constituted "Others". This group is referred to in the text of this paper as other ethnic groups.

^◆^Overall Chi-square p was not significant (p = 0.0774).

Table 3 shows the distribution of in-hospital infant feeding practices. 77.7% initiated breastfeeding within one hour after delivery, 76.5% gave no infant formula, and 84.3% gave no teats while in hospital. There were no statistically significant inter-borough differences in the distribution of these factors.

**Table 3:** Distribution of in-hospital infant feeding practices.

Variable	Total No (%)	BwD No (%)	Hyndburn No (%)	P value
**Breastfeeding initiation**				
Within 1 hr	1,609 (77.7%)	1,212 (77.0%)	397 (79.9%)	0.1748
After 1 hr	462 (22.3%)	362 (23.0%)	100 (20.1%)	
▪ After 1 hr to 48 hrs	419 (20.2%)	329 (20.9%)	90 (18.1%)	0.1765
▪ After 48 hrs	43 (2.1%)	33 (2.1%)	10 (2.0%)	> 0.9999
Total valid data	2,071 (100.0%)	1,574 (100.0%)	497 (100.0%)	-
Missing data	36	32	4	0.0527
**Formula in hospital**				
No formula	1,606 (76.5%)	1,212 (75.8%)	394 (78.8%)	0.1652
Formula	493 (23.5%)	387 (24.2%)	106 (21.2%)	0.1652
▪ Yes (informed choice)	209 (10.0%)	151 (9.4%)	58 (11.6%)	0.1472
▪ Yes (medical indication)	209 (10.0%)	171 (10.7%)	38 (7.6%)	0.0404
▪ Yes (other reasons)	75 (3.6%)	65 (4.1%)	10 (2.0%)	0.0201
Total valid data	2,099 (100.0%)	1,599 (100.0%)	500 (100.0%)	-
Missing data	8	7	1	0.4498
**Teats in Hospital**				
No teats	1,766 (84.3%)	1,349 (84.6%)	417 (83.2%)	0.4399
Yes	329 (15.7%)	245 (15.4%)	84 (16.8%)	0.4399
▪ Yes (informed choice)	159 (7.6%)	95 (6.0%)	64 (12.8%)	< 0.0001
▪ Yes (other reasons)	170 (8.1%)	150 (9.4%)	20 (4.0%)	< 0.0001
Total valid data	2,095 (100.0%)	1,594 (100.0%)	501 (100.0%)	-
Missing data	12	12	0	0.0458

### Infant feeding methods

Table 4 shows the distribution of infant feeding methods up to 6 months. Exclusive breastfeeding rates fell as babies grew older. At every assessment point (i.e. 6 weeks, 17 weeks, and 6 months) exclusive breastfeeding rates were higher in BwD than in Hyndburn. The inter-borough difference in the rates at 6 weeks was not statistically significant (p = 0.0594) but attained statistical significance at 17 weeks (48.2% versus 46.2%; p = 0.0011) and 6 months (14.9% versus 8.5%; p = 0.0002). Mixed breast and formula feeding also declined with increasing age but there were no significant inter-borough differences. Formula feeding peaked at 17 weeks by which time some mothers started introducing weaning foods.

**Table 4:** Infant feeding methods by borough of residence at 6 weeks, 17 weeks and 6 months.

Feeding methods	6 weeks				17 weeks				6 months			
	
	Total No (%)	BwD No (%)	Hyndburn No (%)	P^Δ ^value	Total No (%)	BwD No (%)	Hyndburn No (%)	P^Δ ^value	Total No (%)	BwD No (%)	Hyndburn No (%)	P^Δ ^value
Exclusively breast	1,449 (69.2%)	1,120 (70.3%)	329 (65.8%)	0.0594	930 (46.2%)	740 (48.2%)	190 (39.7%)	0.0011	262 (13.4%)	223 (14.9%)	39 (8.5%)	0.0002

Exclusively formula	327 (15.6%)	235 (14.7%)	92 (18.4%)	0.0484	414 (20.6%)	299 (19.5%)	115 (24.1%)	0.0212	85 (4.3%)	70 (4.7%)	15 (3.3%)	0.1941

Formula & Breast	318 (15.2%)	239 (15.0%)	79 (15.8%)	0.6183	258 (12.8%)	200 (13.0%)	58 (12.1%)	0.5853	55 (2.8%)	43 (2.9%)	12 (2.6%)	0.7511

Breast & solid^#^	0 (0.0%)	0 (0.0%)	0 (0.0%)	-	53 (2.6%)	38 (2.5%)	15 (3.1%)	0.3360	502 (25.6%)	392 (26.2%)	110 (23.9%)	0.0233

Formula & solid^#^	0 (0.0%)	0 (0.0%)	0 (0.0%)	-	4 (0.2%)	4 (0.3%)	0 (0.0%)	0.3111	93 (4.7%)	84 (5.6%)	9 (2.0%)	0.0004

Breast, formula & solid^#^	0 (0.0%)	0 (0.0%)	0 (0.0%)	-	28 (1.4%)	20 (1.3%)	8 (1.7%)	0.3905	179 (9.1%)	130 (8.7%)	49 (10.6%)	0.1972

Solid^#^	0 (0.0%)	0 (0.0%)	0 (0.0%)	-	0 (0.0%)	0 (0.0%)	0 (0.0%)	-	2 (0.1%)	2 (0.1%)	0 (0.0%)	-

Had stopped BF*	0 (0.0%)	0 (0.0%)	0 (0.0%)	-	326 (16.2%)	234 (15.2%)	92 (19.2%)	0.0336	781 (39.9%)	554 (37.0%)	227 (49.2%)	< 0.0001

Total valid data	2,094 (100.0%)	1,594 (100.0%)	500 (100.0%)	-	2,013 (100.0%)	1,535 (100.0%)	478 (100.0%)	-	1,959 (100.0%)	1,498 (100.0%)	461 (100.0%)	-

Missing data	13	12	1	0.2081	94	71	23	0.8057	148	108	40	0.3188

^Δ^p values are for the differences between BwD and Hyndburn mothers.

^#^'Solid' refer to weaning food.

* Little Angels stop follow-up visits/contacts with mothers who had stopped breastfeeding at any assessment point. "Had stopped BF" refers to those mothers.

### Breastfeeding cessation

#### Effects of maternal socio-demographic and obstetric characteristics on breastfeeding cessation

Table 5 shows breastfeeding durations by maternal factors, and the association between these factors and breastfeeding cessation. KM analyses showed that, overall, the mean breastfeeding duration was 21.6 weeks (95% CI 20.86 to 22.37 weeks) and the median duration was 27 weeks (95% CI 25.6 to 28.30 weeks). BwD mothers breastfed significantly longer than Hyndburn mothers. The breastfeeding duration for White mothers was significantly shorter than that for non-White mothers. Among non-White mothers Indians and Blacks breastfed longest while Pakistanis had the shortest duration but the differences were not statistically significant.

**Table 5:** Effects of maternal socio-demographic and obstetric factors on breastfeeding cessation.

	Breastfeeding duration from KM analyses		Unadjusted Cox HR		Adjusted Cox HR^Φ^	
					
Variable	Mean (95% CI)	Median (95% CI)	(95% CI)	P value	(95% CI)	P value
**Borough of residence**						
Both	21.6 (20.86 to 22.37)	27 (25.70 to 28.30)	-	-	-	-
BwD	22.5 (21.63 to 23.39)	27 (26.11 to 27.88)	1^†^	-	-	-
Hyndburn	18.5 (17.36 to 19.73)	22 (18.69 to 25.30)	1.36 (1.18 to 1.55)	< 0.0001	-	-
**Ethnicity**					-	-
White	19.8 (18.53 to 20.97)	20 (17.60 to 22.39)	1^†^	-	1^†^	-
Non-white	23.4 (22.42 to 24.34)	27 (26.03 to 27.97)	0.65 (0.57 to 0.73)	< 0.0001	0.59 (0.52 to 0.67)	<0.0001
▪ Indian	24.8 (23.53 to 26.15)	28 (26.67 to 29.33)	0.55 (0.46 to 0.64)	< 0.0001	0.53 (0.45 to 0.62)	< 0.0001
▪ Pakistani	21.8 (20.27 to 23.25)	26 (25.05 to 26.95)	0.79 (0.68 to 0.91)	0.0015	0.69 (0.60 to 0.81)	< 0.0001
▪ All Blacks	25.1 (20.79 to 29.51)	28 (25.18 to 30.81)	0.40 (0.19 to 0.84)	0.0155	0.35 (0.16 to 0.74)	0.0057
▪ Others^Ψ^	24.5 (20.11 to 28.81)	27 (25.89 to 28.11)	0.52 (0.35 to 0.78)	0.0018	0.48 (0.32 to 0.73)	0.0005
**Marital status**						
Single mothers	18.2 (15.19 to 21.17)	24 (17.77 to 30.23)	1^†^	-	-	-
Mothers with partners	21.6 (20.89 to 22.35)	27 (25.69 to 28.31)	0.80 (0.58 to 1.11)	0.1815	-	-
**IMD quartile**						
1st quartile	21.3 (19.69 to 22.82)	25 (22.71 to 27.29)	1^†^	-	-	-
2nd quartile	20.9 (19.52 to 22.21)	26 (23.36 to 28.64)	1.01 (0.86 to 1.19)	0.8873	-	-
3rd quartile	23.2 (21.37 to 24.97)	27 (26.21 to 27.79)	0.78 (0.66 to 0.92)	0.0043	-	-
4th quartile	20.8 (19.66 to 21.96)	27 (24.86 to 29.13)	0.90 (0.76 to 1.06)	0.2097	-	-
**Parity**						
Primips	20.4 (19.24 to 21.54)	25 (23.19 to 26.81)	1.25 (1.11 to 1.41)	0.0003	1.23 (1.09 to 1.39)	0.0007
Multips^§^	22.5 (21.54 to 23.50)	27 (25.99 to 28.01)	1^†^	-	1^†^	-
▪ 2^nd ^births	20.0 (16.71 to 23.35)	18 (14.21 to21.79)	-	-	-	-
▪ 3^rd ^births	22.4 (21.05 to 23.80)	27 (26.12 to 27.88)	-	-	-	-
▪ 4^th ^births	23.5 (21.55 to 25.36)	27 (23.98 to 30.02)	-	-	-	-
▪ 5^th ^births	22.6 (20.44 to 24.76)	27 (25.63 to 28.37)	-	-	-	-
▪ 6^th ^- 9^th ^births	22.4 (20.46 to 24.42)	29 (not calculated)	-	-	-	-
▪ Mothers with n^th ^birth; (1 ≤ n ≤ 8)	-	-	1†	-	1†	-
▪ Mothers with (n+1)^th ^birth	-	-	0.92 (0.89 to 0.96)	< 0.0001	0.93 (0.89 to 0.97)	0.0006
**Mode of delivery**						
Vaginal	22.0 (21.15 to 22.88)	27 (25.72 to 28.28)	1^†^	-	-	-
▪ Operative delivery	20.4 (18.95 to 21.92)	25 (23.15 to 26.85)	1.16 (1.01 to 1.32)	0.0307	-	-
▪ Caesarean	20.9 (19.10 to 22.71)	25 (23.18 to 26.82)	1.13 (0.96 to 1.31)	0.1336	-	-
▪ Instrumental	19.5 (16.88 to 22.03)	24 (19.67 to 28.32)	1.23 (0.98 to 1.53)	0.0523	-	-

^Φ ^Variables that did not significantly contribute to the fitness of the model were dropped hence no adjusted HRs were calculated for them.

^†^Reference category.

^Ψ^Chinese, Bangladeshi, other ethnic groups constituted "Others". This group is referred to in the text of this paper as other ethnic groups.

^§^Birth order was entered in the Cox model as a continuous variable hence HRs were not reported for the sub-categories used for KM analyses.

Partnered mothers breastfed longer than single mothers. The mean and median breastfeeding durations for multiparae were about 2 weeks longer than those for primiparae. Sub-group analyses showed that 2^nd ^born babies were breastfed for the shortest duration while 4^th ^born babies were breastfed for the longest duration. Mothers delivered vaginally breastfed longer than those who had operative deliveries. Sub-group analysis showed that those who had instrumental deliveries breastfed for the shortest duration. However all these differences were not statistically significant.

Unadjusted Cox regression HR showed that Hyndburn mothers were 36% more likely to stop breastfeeding than BwD mothers (HR: 1.36; 95% CI, 1.18 to 1.55) but the association became insignificant after adjustment for ethnicity. Unadjusted Cox regression HR showed White mothers were 54% more likely to stop breastfeeding compared with non-White mothers (HR: 0.65; 95% CI, 0.57 to 0.73). After adjustment White mothers were 69% more likely to stop breastfeeding compared with non-white mothers (HR: 0.59; 95% CI, 0.52 to 0.67). Sub-group analysis showed that White mothers were 89% more likely to stop breastfeeding compared with Indian mothers (HR: 0.53; 95% CI, 0.45 to 0.62), 45% more likely to stop compared with Pakistanis (HR: 0.69; 95% CI, 0.60 to 0.81) and 2.9 times more likely to stop compared with Blacks (HR: 0.35; 95% CI, 0.16 to 0.81).

Marital status was not associated with breastfeeding cessation in unadjusted Cox regression analyses (HR: 0.80; 95% CI, 0.58 to 1.11). From unadjusted Cox regression analyses primiparae were 25% more likely to stop breastfeeding compared with multiparae (HR: 1.25; 95% CI, 1.11 to 1.41); the association was slightly attenuated after adjustment (HR: 1.23; 95% CI, 1.09 to 1.39). Replacing parity as a binary variable with birth order in the model showed that mothers with babies of higher order birth were less likely to stop breastfeeding than those with lower birth order babies. For example mothers with 3^rd ^born babies were about 8% less likely to stop breastfeeding than those with 2^nd ^born babies (HR: 0.92; p < 0.0001). Unadjusted HR showed that operatively delivered mothers were 16% more likely to stop breastfeeding than mothers delivered vaginally (HR: 1.16; 95% CI, 1.01 to 1.33) and IMD 3^rd ^quartile mothers were 22% less likely to stop breastfeeding than 1^st ^quartile mothers (HR: 0.78; 95% CI, 0.66 to 0.92). However these associations became insignificant after adjustment.

#### Effects of in-hospital infant feeding practices on breastfeeding cessation

Table 6 shows in-hospital infant feeding practices and their effects on breastfeeding cessation. KM analysis showed that there was no statistically significant difference between the duration in mothers who initiated breastfeeding within 1 hour after birth and those who initiated later. Giving babies infant formula and teats in hospital were significantly associated with breastfeeding duration. Mothers who gave infant formula to their babies breastfed for a mean duration of 17.7 weeks (median: 17 weeks) compared with 22.2 weeks (median: 27 weeks) for mothers who did not. The duration varied by the reasons for giving formula: those who gave formula based on informed choice had the shortest duration while those who gave because of medical indications breastfed longest. Mothers who gave teats to their babies breastfed for a mean duration of 17.2 weeks (median: 17 weeks) compared with 22.8 weeks (median: 27 weeks) for those who did not.

**Table 6:** Effects of in-hospital infant feeding practices on breastfeeding cessation.

Variable	Breastfeeding duration from KM analyses		Unadjusted Cox HR	P value	Adjusted Cox HR^Φ^	P value
					
	Mean (95% CI)	Median (95% CI)	(95% CI)		(95% CI)	
**Breastfeeding initiation**						
Within 1 hr	22.2 (21.31 to 23.08)	27 (25.70 to 28.29)	1^†^	-	-	-
After 1 hr	20.3 (18.83 to 21.77)	24 (20.91 to 27.09)	1.24 (1.08 to 1.42)	0.0028	-	-
▪ After 1 hr to 48 hrs	20.3 (18.95 to 21.61)	25 (23.49 to 26.51)	1.20 (1.04 to 1.39)	0.0128	-	-
▪ After 48 hrs	18.0 (13.43 to 22.57)	17 (8.40 to 25.59)	1.52 (1.07 to 2.17)	0.0195	-	-
**Formula in hospital**						
No formula	22.8 (21.86 to 23.68)	27 (26.08 to 27.92)	1^†^	-	1^†^	-
Formula	17.7 (16.53 to 18.89)	17 (13.94 to 20.06)	1.56 (1.36 to 1.78)	< 0.0001	1.50 (1.26 to 1.79)	< 0.0001
▪ Yes (informed choice)	19.7 (17.56 to 21.87)	20 (14.40 to 25.59)	1.73 (1.44 to 2.07)	< 0.0001	1.91 (1.49 to 2.44)	< 0.0001
▪ Yes (medical indication)	16.6 (14.93 to 18.18)	17 (13.79 to 20.20)	1.38 (1.14 to 1.68)	0.0009	1.34 (1.09 to 1.65)	0.0058
▪ Yes (other reasons)	17.0 (14.54 to 19.50)	18 (9.31 to 26.39)	1.62 (1.20 to 2.18)	0.0016	1.53 (1.07 to 2.17)	0.0181
**Teats in Hospital**						
No teats	22.4 (21.56 to 23.26)	27 (26.17 to 27.82)	1^†^	-	1^†^	-
Yes	17.2 (15.88 to 18.51)	17 (14.42 to 19.58)	1.61 (1.38 to 1.87)	< 0.0001	1.26 (1.04 to 1.53)	0.0203
▪ Yes (informed choice)	17.3 (15.52 to 19.14)	18 (13.65 to 22.35)	1.51 (1.23 to 1.87)	0.0001	1.01 (0.77 to 1.33)	0.9231
▪ Yes (other reasons)	17.2 (15.35 to 19.05)	17 (13.94 to 20.05)	1.69 (1.39 to 2.06)	< 0.0001	1.32 (1.04 to 1.68)	0.0221

^Φ ^Variables that did not significantly contribute to the fitness of the model were dropped hence no adjusted HRs were calculated for them.

^†^Reference category.

Unadjusted Cox regression results suggested that initiating breastfeeding after 1 hour post-delivery increased the likelihood of breastfeeding cessation but the association became insignificant after adjustment. Unadjusted HRs showed that mothers who gave infant formula while in hospital were 56% more likely to stop breastfeeding compared with those who did not (HR: 1.56; 95% CI, 1.36 to 1.78). The association was slightly attenuated after adjustment (HR: 1.50; 95% CI, 1.26 to 1.79). Adjusted sub-group analyses results also showed that mothers who gave infant formula based on informed choices were most likely to stop breastfeeding (HR: 1.91; 95% CI, 1.49 to 2.44) while those who gave it based on medical indications were least likely to stop (HR: 1.34; 95% CI, 1.09 to 1.65). A similar association was found for the use of teats in hospital. Unadjusted Cox regression results showed that mothers who gave teats while in hospital were 61% more likely to stop breastfeeding compared with those who did not (HR: 1.61; 95% CI, 1.38 to 1.87). The association was markedly attenuated after adjustment (HR: 1.26; 95% CI, 1.04 to 1.53).

## Discussion

Our study showed that ethnicity and parity were independent predictors of breastfeeding cessation. The UK 2005 Infant Feeding Survey and other studies have shown that White mothers breastfeed for shorter durations compared with mothers from other ethnic groups [3,21,37]. Among mothers from Asian background in the UK Pakistani and Bangladeshi mothers stop breastfeeding sooner than Indian mothers [23]. Many studies showed that breastfeeding duration increases with increasing parity [3,26,38,39] and some evidence suggests that this might be related to previous breastfeeding experiences [3,22].

Our study also found that giving infant formula and/or teats to babies in hospital were significantly associated with breastfeeding cessation. These in-hospital infant feeding practices, especially when based on informed choices, may not in themselves be predictive of breastfeeding cessation but may be the early manifestations of maternal intentions relating to long-term infant feeding practices. The literature is consistent about the detrimental effects of using teats on breastfeeding outcomes [13,29-33] for which there may be a physiological explanation. The innate sucking reflex of the infant is satisfied by the teat, decreasing or eliminating the desire for contact with the nipple leading to reduction in breast milk production [13,30]. However others have suggested that teat use may simply be a marker for socio-demographic, psychosocial and cultural factors that determine both teat use and breastfeeding [30].

Our results suggested that the reasons for giving formula and/or teats may influence breastfeeding duration. Mothers who gave formula based on informed choice were most likely to stop breastfeeding. The timing of the decision to breastfeed influences breastfeeding duration [24] and it is likely this sub-group of mothers had not decided to breastfeed their babies early during the antenatal period. Those who gave formula for medical indications were least likely to stop breastfeeding and this was consistent with the WHO findings [13].

We found in our study that known predictors of breastfeeding cessation such as marital status, socio-economic deprivation, mode of delivery, and the timing of breastfeeding initiation, were not associated with breastfeeding cessation suggesting the peer support these women received might have been beneficial in attenuating the effects of these factors. Partnered mothers are usually more likely to breastfeed longer than single mothers [24-28] and in the developed countries affluent mothers tend to breastfeed longer [3,4,24,29]. Caesarean delivery is a recognised risk factor for breastfeeding initiation failure but there is no consistent evidence on its effect on breastfeeding duration [24,25]. Recent evidence has suggested that operative deliveries may have detrimental effects on breastfeeding outcomes [40-43] probably mediated by delayed breastfeeding initiation [40,44] as a possible consequence of anaesthesia and analgesia [45,46]. However the UK 2005 Infant Feeding Survey found that mothers were equally likely to be breastfeeding at one and two weeks regardless of delivery methods [3]. Our study has found that mode of delivery was not associated with breastfeeding cessation supporting the conclusion by some that if the cultural and hospital environments generally promote breastfeeding, operative deliveries have little negative impact on breastfeeding outcomes [47].

Delayed breastfeeding initiation adversely affects breastfeeding duration and this formed the basis for the WHO/UNICEF recommendation that breastfeeding should be initiated within 30 minutes of birth even though it was admitted it is difficult to make exact recommendation about the timing of initiation [13]. In our study 77.7% of the mothers had initiated breastfeeding within one hour after delivery broadly in keeping with the recommendation but this was not associated with breastfeeding cessation.

### Implications

Our study is one of the few to examine the effects of maternal factors and early infant feeding practices in hospital on breastfeeding in a peer support setting in the UK. The results have suggested that infant feeding practices associated with maternal ethnicity and parity may be more difficult to influence by peer support interventions. Peer support programmes, particularly those in multi-ethnic settings, will need to identify the needs of their various client groups in order to appropriately support them breastfeed longer. This could be done through an effective evaluation of such programmes.

We have also identified that in-hospital infant feeding practices which are likely to continue after leaving hospital have a negative impact on breastfeeding continuation. Infant feeding practices are influenced by the socio-cultural milieu and maternal intentions relating to these practices tend to be consolidated during the ante-natal period. For peer support programmes to be more effective we suggest their support start during this period to enable mothers form healthier infant feeding intentions.

### Strengths, limitations and biases

In this study we used a routinely collected data. Problems of a routine data source are largely related to completeness and accuracy. Missing dates of breastfeeding cessation affected BwD more than Hyndburn. Missing data resulted in 2,074 records out of 2,107 to be included in the final Cox multiple regression model. These made the study prone to selection bias. A potential confounder not included in the analyses was maternal age because this was not collected in many cases. An additional shortcoming relates to the accuracy of the estimated breastfeeding duration based on the on self-reported dates of breastfeeding cessation and infant feeding methods.

The strength of our study lies in the statistical methods used. The use of survival analysis to determine the breastfeeding duration provides a good understanding of breastfeeding behaviour over time [27]. Furthermore this type of analysis is suitable for data collected from follow-up studies that involve censoring and/or in which the time to an event is regarded as the outcome variable of interest [36]. To quantify the association between the factors and breastfeeding cessation Cox proportional hazards model was used. This model allows joint estimation of the effects of predictor variables on the risk for breastfeeding cessation rather than the duration itself [27].

## Conclusion

In this study ethnicity, parity and in-hospital infant feeding practices remained independent predictors of breastfeeding cessation in this peer support setting. However other recognised predictors of breastfeeding cessation such as marital status, mode of delivery, and socio-economic deprivation were not found to be associated with breastfeeding cessation.

## Competing interests

GA was involved in evaluating Little Angels peer support for breastfeeding mothers in Hyndburn. EJ is one of the Directors of Little Angels. EM was the acting Director of Public Health at Hyndburn and Ribble Valley PCT (now part of NHS East Lancashire) at the time of this study. Little Angels is funded in part by NHS East Lancashire and Blackburn with Darwen Borough Council.

## Authors' contributions

All the authors contributed to the various stages of this study. EM conceived the idea and liaised with Little Angels. EJ designed the data collection tool and supervised the data collection. GA did the analysis and wrote the paper with AV. All the authors read and commented on the drafts and approved of the final version for submission.

## Pre-publication history

The pre-publication history for this paper can be accessed here:

http://www.biomedcentral.com/1471-2431/10/3/prepub
